# Clinical Utilization Pattern of Liquid Biopsies (LB) to Detect Actionable Driver Mutations, Guide Treatment Decisions and Monitor Disease Burden During Treatment of 33 Metastatic Colorectal Cancer (mCRC) Patients (pts) at a Fox Chase Cancer Center GI Oncology Subspecialty Clinic

**DOI:** 10.3389/fonc.2018.00652

**Published:** 2019-01-17

**Authors:** Pooja Ghatalia, Chad H. Smith, Arthur Winer, Jiangtao Gou, Lesli A. Kiedrowski, Michael Slifker, Patricia D. Saltzberg, Nicole Bubes, Fern M. Anari, Vineela Kasireddy, Asya Varshavsky, Yang Liu, Eric A. Ross, Wafik S. El-Deiry

**Affiliations:** ^1^Department of Hematology/Oncology, Fox Chase Cancer Center, Philadelphia, PA, United States; ^2^Department of Biostatistics and Bioinformatics, Fox Chase Cancer Center, Philadelphia, PA, United States; ^3^Guardant Health, Redwood City, CA, United States

**Keywords:** liquid biopsy, precision oncology, molecular target, tumor heterogeneity, drug resistance, tumor burden, cfDNA

## Abstract

**Background:** Liquid biopsy (LB) captures dynamic genomic alterations (alts) across metastatic colorectal cancer (mCRC) therapy and may complement tissue biopsy (TB). We sought to describe the utility of LB and better understand mCRC biology during therapy.

**Methods:** Thirty-three patients (pts) with mCRC underwent LB. We used permutation-based *t*-tests to assess associations between alts, and clinical variables and used Kendall's tau to measure correlations.

**Results:** Of 33 pts, 15 were women; 22 had colon, and the rest rectal cancer. Pts received a median of two lines of therapy before LB. Nineteen pts had limited testing on TB (*RAS/RAF/TP53/APC*), 11 extended NGS, and 3 no TB. Maxpct and alts correlated with CEA (*p* < 0.001, respectively). In 3/5 pts with serial LB, CEA correlated with maxpct trend, and CT tumor burden. In 6 pts, mutant *RAS* was seen in LB and not TB; 5/6 had received anti-EGFR therapy prior to LB, suggesting *RAS* alts developed post-therapy. In two pts *RAS*-mutated by TB, no *RAS* alts were detected on LB; these pts had low disease burden on CT at time of LB that also did not reveal *APC* or *TP53* alts. In six patients who were KRAS wt based on TB, post anti-EGFR LB revealed subclonal KRAS mutations, likely a treatment effect. The median number of alts was higher post anti-EGFR LB (*n* = 12) vs. anti-EGFR nave LB (*n* = 22) (9.5 vs. 5.5, *p* = 0.059) but not statistically significant. More alts were also noted in post anti-EGFR therapy LB vs. *KRAS* wt anti-EGFR-nave LB (*n* = 6) (9.5 vs. 5) among patients with *KRAS* wild-type tumors, although the difference was not significant (*p* = 0.182).

**Conclusions:** LB across mCRC therapy detects driver mutations, monitors disease burden, and identifies sub-clonal alts that reflect drug resistance, tumor evolution, and heterogeneity. Interpretation of LB results is impacted by clinical context.

## Introduction

A key factor contributing to the lethal outcome of cancer, therapeutic failure, and drug resistance is intra-tumoral heterogeneity and clonal evolution of tumors caused by accumulation of somatic mutations (1,3). The advent of next-generation sequencing has enabled more powerful analysis of tumor evolution and has improved our understanding of tumor initiation and development (1, 2, 4, 5). In patients with advanced colorectal cancer who receive multiple lines of therapy during the course of treatment, understanding the evolution of genetic alts during treatment can inform clinical management, and clinical trial design (6,9). This is becoming important in the era of precision oncology where acquired mutations may suggest novel options for therapy or resistance to targeted agents (10,12).

Guardant360 is an assay that utilizes next generation sequencing of cell-free DNA (cfDNA) to comprehensively profile 73 cancer-related genes in peripheral blood to establish circulating tumor (ct)-DNA presence, mutation patterns, and quantity (13). Multiple validation studies have been published utilizing this assay, including analytical studies and clinical validations in patients with advanced non-small cell lung cancer, colorectal cancer, and other solid tumors; such studies demonstrate high concordance between clinical plasma- and tissue-based genotyping methods which supports the clinical accuracy of the Guardant360 LB assay (3, 14, 15). An analysis of the landscape of cfDNA alts detected in a large cohort of colorectal cancer patient samples analyzed with this assay showed high similarity with genomic alts from tissue studies (7).

Here we present a case series of patients with advanced colorectal cancers who underwent LB testing at various time points during the course of their treatment at a Fox Chase Cancer Center GI Oncology Clinic. We describe our experience and delineate the utility of LB in practice and try to better understand the biology of metastatic colorectal cancer. Our findings point to the utility of LB in clinical practice during the care of patients with advanced colorectal cancer at an academic NCI-designated comprehensive cancer center. They suggest the importance of clinical context with regard to interpretation of LB test results, and illustrate uses, and information gained beyond which specific mutations are detectable. LB can reveal changes in tumor burden with ongoing therapy, a range of sub-clonal mutations likely due to acquired drug resistance, and clinical insight into tumor heterogeneity. Our retrospective case study was not designed to allow for clinical practice recommendations but rather to demonstrate preliminary clinical use patterns at an academic GI cancer clinic such that in the future specific uses or outcomes of interest can be further investigated.

## Methods

### Patient Selection

Patients who underwent LB from Jan 2016 to April 2018 in a single colon cancer specialty clinic (W.S.E-D.) at Fox Chase Cancer Center were identified and studied. This was an institutional review board (IRB)-approved retrospective study. Clinical information including date of diagnosis, age at diagnosis, gender, type of cancer: colon vs. rectal, stage at diagnosis, lines of therapy, date of LB, reason for LB, date of tissue biopsy, CEA at time of LB and tissue biopsy, tumor burden on CT scan at time of LB and tissue biopsy, and last date of follow-up were recorded.

### Genomic Testing

All patient samples were collected and processed in accordance with the Guardant360 clinical blood collection kit instructions (Guardant Health, Inc.). Guardant360 interrogates cfDNA for single nucleotide variants (SNVs) in 73 cancer-related genes, indels in 23 genes, copy number amplifications (CNAs) in 18 genes, and fusions in six genes. A routine blood draw (two, 10-mL Streck tubes) was obtained in the clinic and sent to Guardant Heath, a Clinical Laboratory Improvement Amendments (CLIA)-licensed, College of American Pathologists-accredited, New York State Department of Health-approved clinical laboratory. No refrigeration or local centrifugation was needed. For each sample, cfDNA was extracted from stabilized whole blood and between 5 and 30 ng of cfDNA input per sample was analyzed as described previously (3, 13). While the input was 530 ng of extracted ctDNA, ~2/3 of the samples used 30 ng, but the minimum required was 5 ng of extracted ctDNA. In brief, DNA fragments were labeled at high efficiency with non-random oligonucleotide adapters (molecular barcodes), and used to prepare sequencing libraries, which were then enriched using hybrid capture and sequenced. Sequencing reads were then used to reconstruct individual cfDNA molecules present in the original patient sample with high fidelity using proprietary double-stranded consensus sequence representation. From the LB report, genomic alts, type of alts, and somatic alteration burden (maxpctdefined as the percentage frequency of the alteration with highest mutant allele frequency reported in the sample) were recorded.

### Tissue Biopsy

For patients who had undergone tissue biopsy during their clinical course, the genes tested, and mutations identified in the tissue biopsy were recorded. Some patients with tissue biopsy had a restricted panel of next-generation sequencing (NGS) testing for alts in *RAS, RAF, TP53*, and/or *APC*, while others had more extensive testing using various commercially available NGS test panels (FoundationOne, Caris, Nanthealth, Tempus, Omniseq).

### Statistical Analysis

We used Kendall rank correlation tau to measure associations between continuous variables. For comparing numbers of alts between groups, we applied permutation-based two-sample *t*-tests. When laboratory measurements were available from multiple time points for the same patient we selected data from the blood sample collected at time of disease progression for this comparison. All tests were two-sided with a 5% type I error. Data were analyzed in statistical software R (version 3.5.0) and SAS (version 9.4).

## Results

### Clinical Characteristics/Patient Demographics

Of 33 patients, 11 patients had rectal tumors and 22 had colon cancer. There were 18 women and 15 men. Median age at diagnosis was 52 years (range 2076). Four patients initially presented with stage 2 disease, 9 patients with stage 3 disease, and 20 patients presented with metastatic disease at diagnosis. All patients were metastatic at the time of LB. Patients received a median of two (range 07) lines of therapy before LB.

### Tissue and Liquid Biopsy Characteristics

**Figure 1:**
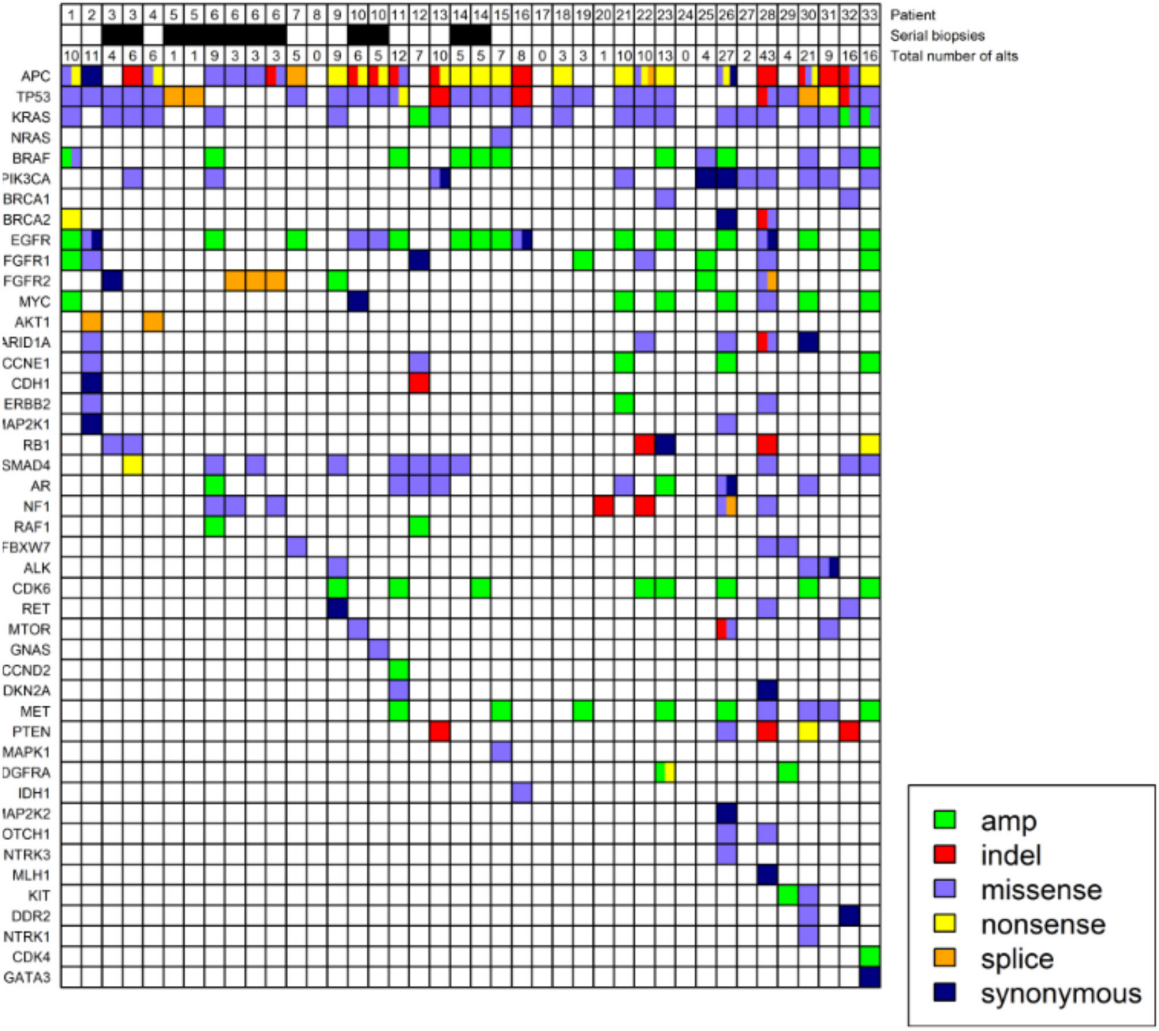
Genomic profiling of circulating free tumor DNA in 33 patients with metastatic colorectal cancer. Four patients underwent serial LB as indicated in the second row. The number of alts detected on LB are listed in the third row. Colors denote different types of alts. Allele frequencies associated with the alts can be found in Supplementary Table 1.

Nineteen patients had limited tumor tissue NGS, 11 patients had extensive tumor NGS testing, and 3 patients had no NGS on tumor tissue. Eighteen patients were *KRAS* wild-type and 12 patients were *KRAS* mutant (Figure 1). One patient's tumor was MSI-high, 13 patients had microsatellite stable tumors, and 9 patients had not had MSI testing on the tumor. All detected mutations and their allele frequencies are listed in Supplementary Table 1.

In 5 patients LB was obtained due to inability to obtain tissue biopsy and in 28 patients to assess mutation load/identify targetable alts. Five patients had serial LBs. Of these, 1 patient had LB four times and the rest had LB twice during their treatment course. When including all LB results, including serial LB, median maxpct was 11.6% (range 083.9%). The median number of alts detected was 6 (range 043). Median CEA at the time of LB was 56 (range 14090). The median time from blood draw to obtaining results for LB was 14 days.

### Correlation Between Tumor Burden and LB Alts

**Figure 2:**
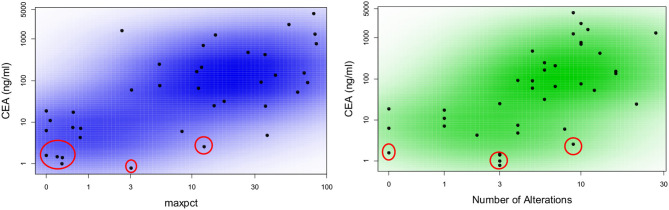
Correlation between CEA as a marker of tumor burden and number of alterations or maximal allele frequency found in liquid biopsy from patients with metastatic colorectal cancer. Scatter plot colored by smoothed density demonstrating correlation between maxpct and number of alts with CEA. Plots include data from all available blood samples. There is a direct correlation between maxpct with CEA (Kendall's Tau = 0.436; *p* = 0.001) and number of alts with CEA (Kendall's Tau = 0.451; *p* < 0.001). The values circled in red represent three patients who never had elevated CEA despite high burden of metastatic disease. One of these patients had four serial liquid biopsies.

**Figure 3:**
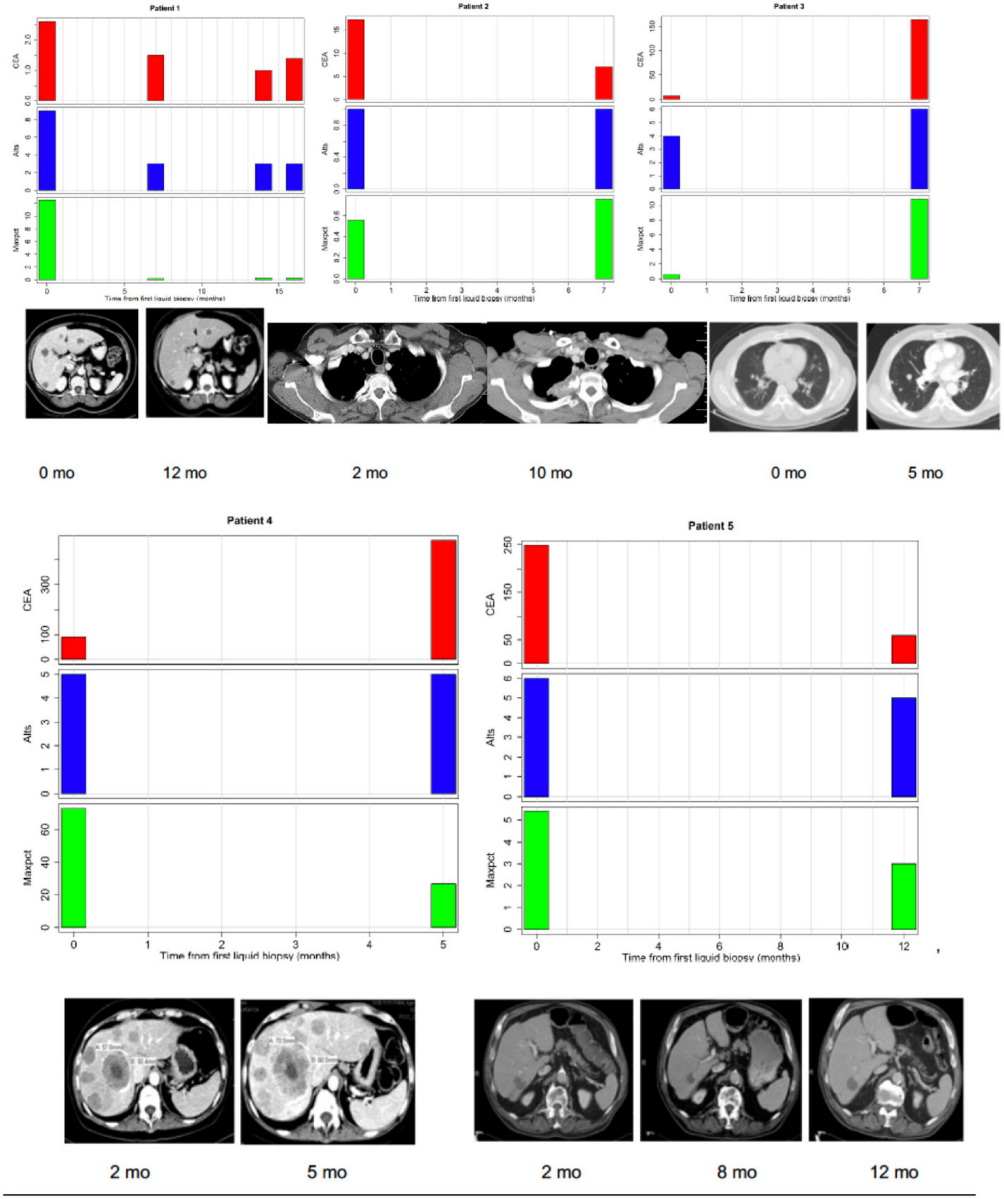
Correlations between tumor burden as assessed by radiographic imaging (CT scans) or CEA (tumor marker) and liquid biopsy mutation parameters (alts or number of alterations/number of mutated genes and maxpct or maximal allele frequency of mutated allele). **(Top Left)** Maxpct, CEA, and alts follow a downward trend as disease on CT scan improves. Center: With growth of mediastinal mass on CT, note rise in maxpct, CEA, and alts. **(Top Right)** As lung disease worsens on CT, maxpct, CEA, and alts increase. **(Bottom Left)** Despite increasing tumor on CT scan and rising CEA, maxpct did not rise. Liquid biopsy did contain APC and TP53 mutations, indicating presence of ctDNA. **(Bottom Right)** Liver metastases decreased between 1/2017 and 7/2017 and then increased in 11/2017. Allele freq. low (24%) probably due to low disease burden on CT.

We hypothesized that LB results obtained at a given time might provide representative information on tumor burden, and may correlate with other measures appropriate to the clinical context. CEA is a good surrogate blood-based marker for tumor burden in most patients with colorectal cancer. In three patients (one of whom had four serial biopsies) CEA was always normal (<3 ng/ml) despite a high tumor burden indicating that their tumor did not produce CEA (Figure 2). We found that CEA correlated positively with maxpct (Kendall's Tau = 0.436, *p* = 0.001) and number of alts (Kendall's Tau = 0.451, *p* < 0.001) present in the LB consistent with our hypothesis (Figure 2). One of three pts with 0 LB alts detected had no measurable disease on CT scan, and the others had several lesions noted on CT scan. In the other 2 patients, LB did not have *APC, p53, KRAS*, or *PIK3CA* mutations, which may suggest that the LB specimen may not have captured any ctDNA. In 3/5 pts with serial LBs, CEA correlated with maxpct trend and CT tumor burden (Figure 3). In one of the patients in whom the CEA, number of alts and maxpct did not correlate with CT tumor burden, the allele frequency was very low (24%) and is probably due to low disease burden on CT. In another patient with an unexpected pattern of the LB results and CT scan tumor burden, necrosis demonstrated in the CT scan may have led to unreliable ctDNA results. Relationships between tumor burden and findings of LB results, including the presence or absence of accompanying tumor gene mutations involved in CRC, provided an indication that the clinical context in which an LB was performed would ultimately impact the results and their interpretation.

### Liquid Biopsy to Guide Treatment Decision

We hypothesized that the presence of *RAS* mutations in LB may not only guide treatment decision for mCRC but might also reveal developing subclonal mutations in mCRC patients receiving anti-EGFR therapy. In two patients, due to insufficient tissue available for testing, *RAS* status could not be determined. LB obtained at the time of disease progression ultimately revealed clear evidence for *KRAS* mutation and anti-EGFR therapy was avoided in this patient. In another patient referred from an outside institution, *RAS* testing was not sent on tissue biopsy. LB in this patient revealed KRAS mutation and thus anti-EGFR therapy was not recommended as a first-line treatment option. In another patient, serial LBs revealed *KRAS* G12D mutation in both instances with allele frequency 0.49 and 6.46%, respectively (patient three in Figure 1). However, a tissue biopsy obtained at a time point between the two LB did not reveal *RAS* mutations, likely an example of tumor heterogeneity. In this patient, anti-EGFR therapy was avoided. LB can overcome some shortcomings of tissue biopsy, as a complementary test.

### Association Between Number of Alts and Anti-EGFR Therapy

**Figure 4:**
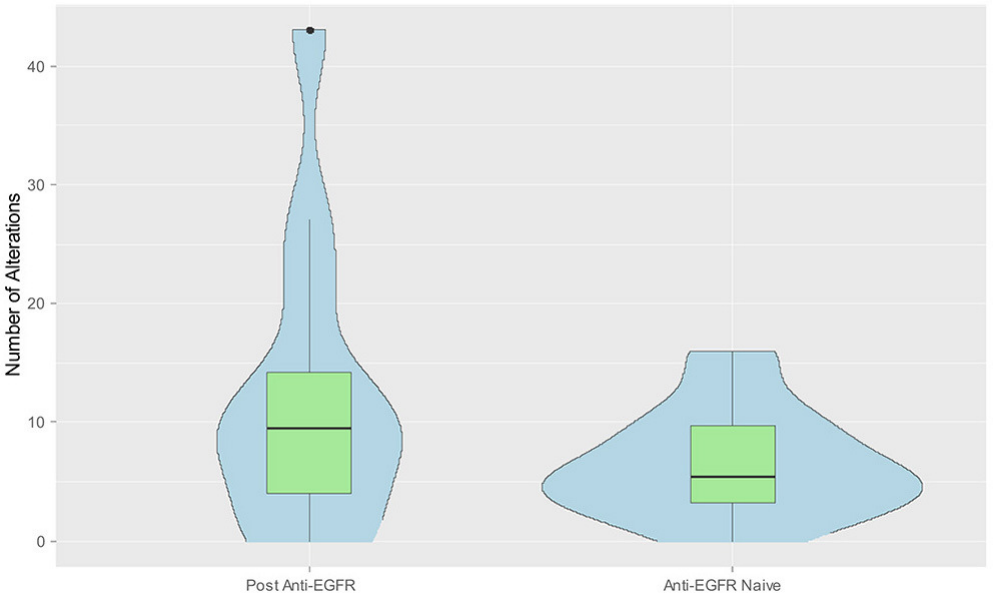
Combined violin and box plot graph demonstrating increased number of alts in liquid biopsy post anti-EGFR therapy. Data from the blood sample collected at time of disease progression was selected when multiple measurements were available from the same patient. Median number of alts were higher post anti-EGFR (*n* = 12) vs. anti-EGFR nave liquid biopsy (*n* = 22) (9.5 vs. 5.5, *p* = 0.058).

We hypothesized that anti-EGFR therapy might increase the number of genomic alts in the tumor. We compared the median number of alts in LBs of patient's pre- and post-anti-EGFR therapy. Overall, the median number of alts was higher post anti-EGFR (*n* = 12; median = 9.5) vs. anti-EGFR nave (*n* = 22; median = 5.5) LB, however the difference did not quite reach the pre-defined level for statistical significance (*p* = 0.059) (Figure 4). Likewise, among patients with *KRAS* wild-type tumors more alts were noted in post anti-EGFR therapy LB (*n* = 12; median = 9.5) vs. anti-EGFR nave (*n* = 6; median = 5) LB. This difference was not statistically significant (*p* = 0.182). Of note, in six patients who were KRAS wt based on tissue biopsy, post anti-EGFR subclonal KRAS mutations developed, and is likely an effect of treatment. One patient post-cetuximab anti-EGFR therapy had five KRAS gene mutations (A146T^*^, G12V, G13D, G60R, K117N), 3 EGFR alterations (N493D, P373S, Y1172Y), 7 p53 mutations (A161T, C176R, Q354R^*^, R282W, p.Lys382fs, p.Ser2fs^*^, p.Val73fs), among others. Some of the mutations were present at fairly high allele frequencies, with an ^*^ indicating allele frequency >4%. The value of 4% allele frequency was set empirically for one case to highlight specific enriched alleles in that case where there were multiple mutations in a number of driver and drug-resistance genes found post-cetuximab therapy. The value is on the high end to show that for example in the case of the KRAS gene there was a dominant allele (A146T) and multiple other less frequent alleles likely reflecting the tumor's heterogeneity and the subclonal nature of the mutations. In a recently published large clinical sample set using the guardant technology (~21,000 patient samples) the median mutant allele fraction for alterations was 0.4% and the mean was 3.67% (16), and so 4% would be on the higher end of the spectrum. Thus, patients receiving anti-EGFR therapy can develop a high number alterations found in LB that likely represent acquired resistance mechanisms, e.g., multiple sub-clonal KRAS mutations.

## Discussion

Our results provide a clinically and genomically annotated case series in mCRC detailing the clinical experience with LB results in a cohort receiving systemic chemotherapy combinations, and includes several patients who underwent serial LB. In CRC, the most common mutations are *APC* (incidence 80%), *TP53* (50%), all *RAS* (40%), *BRAF* (810%), and *PIK3CA* (12%) (17,20). These were also the most common mutations identified on LB in this cohort (Figure 1). For LB results with complete absence of these or any other mutations, there is a need for caution in interpretation as the results may indicate lack of sufficient ctDNA. For example, LB in patients 8, 17, 20, 24 in Figure 1 may not have had sufficient ctDNA, and the lack of alts on LB may not be representative of *RAS* status. With very low or absent disease burden, it would be expected that cfDNA may not be measurable. The expected profile of genomic alts (which genes are mutated) will vary among patients with different tumor types based on the observed common drivers. Our results provide insight into what is observed in a typical cohort of mCRC patients.

In 10 patients, there was discordance between the results of *KRAS* mutation in LB and tumor tissue. Importantly, the tissue biopsy and LB were obtained at different time points in all of these cases, and thus tumor evolution or therapy effects could have impacted the results and may explain the discordance. Moreover, in 6 of these 10 cases, LB obtained after anti-EGFR therapy revealed subclonal *KRAS* mutations that were not identified in the tissue biopsy obtained earlier. In two patients, *KRAS* mutation present in TB was absent in LB obtained later. One of these patients had minimal disease on CT scan at the time of LB indicating possible lack of ctDNA. LB also did not reveal *APC* or *TP53* alts in this patient, suggesting undetectable tumor-derived cfDNA overall. One patient with *KRAS* mutation on TB had serial LBs. The first LB showed *KRAS* mutation and the subsequent 3 LBs showed no *KRAS* mutations. This is likely due to the patient's significant tumor response observed on CT scan between the first and subsequent LBs. Of note, this patient did not receive any anti-EGFR therapy. Our results point to the importance and relevance of clinical context with regard to interpretation of LB test results, and further illustrate uses and information gained beyond just which specific gene mutations are detected in cfDNA analysis. LB clearly reveals changes in tumor burden with ongoing therapy, a range of sub-clonal mutations most likely due to acquired drug resistance, and clinical insight into tumor heterogeneity. This includes heterogeneity post-therapy exposure.

One patient with an MSI-H tumor demonstrated on TB testing had 11 alts on LB (patient two in Figure 1), while a patient with 27 alts was MSS (patient 26 in Figure 1), and MSI status was not determined in a patient with 43 alts on LB (patient 28 in Figure 1). We suspect that patients who were heavily pretreated acquired a significant number of gene mutations that may promote cell survival and resistance to chemotherapy or targeted therapy.

Clonal hematopoeisis (CH) is the somatic acquisition of genomic alts in hematopoietic stem and/or progenitor cells, leading to clonal expansion (21). In patients with cancer, CH is a common occurrence, associated with aging, smoking, and radiation therapy (22). CH is associated with increased risk of therapy-related hematologic malignant neoplasms, and genes frequently mutated in CH such as *DNMT3, TET2, PPM1* are also commonly altered in hematologic malignant neoplasms (23). While CH mutations in both tissue and LB may be misattributed as somatic tumor variants in patients, the Guardant360 panel did not test for mutations in these frequently mutated CH genes. While *TP53* or *KRAS* can be associated with CH, we believe that clonal hematopoiesis likely has a minimal role in this study as the objective of obtaining LBs in this cohort was to identify therapy-related resistance mechanisms during treatment course and to monitor tumor burden as a measure of therapy response by serial LBs.

In our case series, we show that LBs can help identify actionable driver mutations, guide treatment decisions, and monitor disease burden. LBs offer several advantages over TB. In addition to being non-invasive, LBs can be much more easily performed serially during treatment course, can help monitor disease burden, treatment effect, and developing resistance mutations, and can detect tumor heterogeneity that is a limitation with use of tissue biopsy. Our results provide clinical experience with use of this technology in a limited mCRC cohort at an academic center and illustrate how information may be used to impact clinical decision-making. However, larger studies are needed to address any recommendations that may impact on clinical practice. In addition, insights were gained regarding the biology of treatment response and resistance. LB appears to have some clinical utility in the ongoing care of patients with mCRC including the timely identification of *RAS* family gene mutations, and understanding the basis for emerging resistance to anti-EGFR therapy. An important insight gained from our experience is that clinical context matters and can have an impact on the interpretation of LB results. While we believe CH had minimal impact on the uses we describe for LB in our cohort, this is clearly an important consideration that could have impact on the interpretation of LB results especially in certain contexts with rare sub-clonal alts whose origin may not be the patient's tumor. For mCRC patients, the presence or absence of tumor Ras mutations directly impacts on use of anti-EGFR therapy. Thus, it is critically important to have valid information for the Ras gene family status with appropriate interpretation taking into account the possible confounder of effects of clonal hematopoiesis. This suggests that the input of experts in cancer genetics and molecular tumor boards (as well as potentially testing of normal WBC DNA in specific situations) may ultimately enhance the clinical utility of LB in patient care.

In conclusion, based on our experience we suggest considering LB for patients who have not had TB or have insufficient tissue to determine the presence of *KRAS/NRAS/BRAF* mutations, especially in the context of high tumor burden prior to therapy. LB could also be considered in patients who do not have an elevated CEA as serial LB may help monitor disease response during treatment. In patients who appear to have no evidence of disease after therapy, periodic LB during surveillance period may help detect disease relapse if alts are detected. LB may also help monitor the evolution of resistance mechanisms in the tumor and recent data indicate that LB results may allow re-challenge of previously received anti-EGFR therapy (24). In this study the authors note that in *RAS/RAF/EGFR* wild-type patients progressing on anti-EGFR therapy, the clones of *RAS and EGFR*, as detected on LB exponentially decay and knowing the half-life of these clones can help predict the efficacy of re-challenging these patients with anti-EGFR therapy (24). Larger studies in the future need to more definitively establish the ability of liquid biopsies to safely substitute for tissue biopsies in certain clinical settings for mCRC patients and to determine the optimal frequency of obtaining LB in different clinical settings. Our study was a retrospective case series that was neither designed nor intended to make clinical practice recommendations but to motivate larger studies that are statistically powered to allow for specific recommendations in different clinical situations where use of liquid biopsy may be advantageous in the clinical care of patients. However, LB as an adjunct, complementary technology appears to have some utility in the monitoring and treatment decisions for patients with advanced mCRC especially in settings where TB results are unavailable, not possible or impractical.

## Ethics Statement

Upon review, the IRB at Fox Chase Cancer Center determined the submission (IRB# 17-9057: Pilot Analysis of Utility of Liquid Biopsy Results in Advanced Colorectal Cancer) meets the criteria for the approval of research outlined in 45 CFR 46.111. Upon review, the IRB determined the submission meets the criteria for Waiver of Informed Consent outlined in 45 CFR 46.116(d). Upon review, the IRB determined the submission meets the criteria for Waiver of Authorization for Use or Disclosure outlined in 45 CFR 164.512(i)(2). The IRB approved the protocol from 1/12/2018 to 1/11/2019 inclusive.

## Author Contributions

All authors listed have made a substantial, direct and intellectual contribution to the work, and approved it for publication.

### Conflict of Interest Statement

The authors declare that the research was conducted in the absence of any commercial or financial relationships that could be construed as a potential conflict of interest.
