# Corrigendum: Clinical Utilization Pattern of Liquid Biopsies (LB) to Detect Actionable Driver Mutations, Guide Treatment Decisions and Monitor Disease Burden During Treatment of 33 Metastatic Colorectal Cancer (mCRC) Patients (pts) at a Fox Chase Cancer Center GI Oncology Subspecialty Clinic

**DOI:** 10.3389/fonc.2021.674782

**Published:** 2021-05-07

**Authors:** Pooja Ghatalia, Chad H. Smith, Arthur Winer, Jiangtao Gou, Lesli A. Kiedrowski, Michael Slifker, Patricia D. Saltzberg, Nicole Bubes, Fern M. Anari, Vineela Kasireddy, Asya Varshavsky, Yang Liu, Eric A. Ross, Wafik S. El-Deiry

**Affiliations:** ^1^ Department of Hematology/Oncology, Fox Chase Cancer Center, Philadelphia, PA, United States; ^2^ Department of Biostatistics and Bioinformatics, Fox Chase Cancer Center, Philadelphia, PA, United States; ^3^ Guardant Health, Redwood City, CA, United States

**Keywords:** liquid biopsy, precision oncology, molecular target, tumor heterogeneity, drug resistance, tumor burden, cfDNA

In the original article, there was a mistake in **Figure 3** as published. Incorrect CT scans were provided for two patients, while another patients CT scan was erroneously duplicated. The error was previously missed by the authors and the reviewers.

The corrected **Figure 3** appears below.

The authors apologize for this error and state that this does not change the scientific conclusions of the article in any way. The original article has been updated.

**Figure 3:**
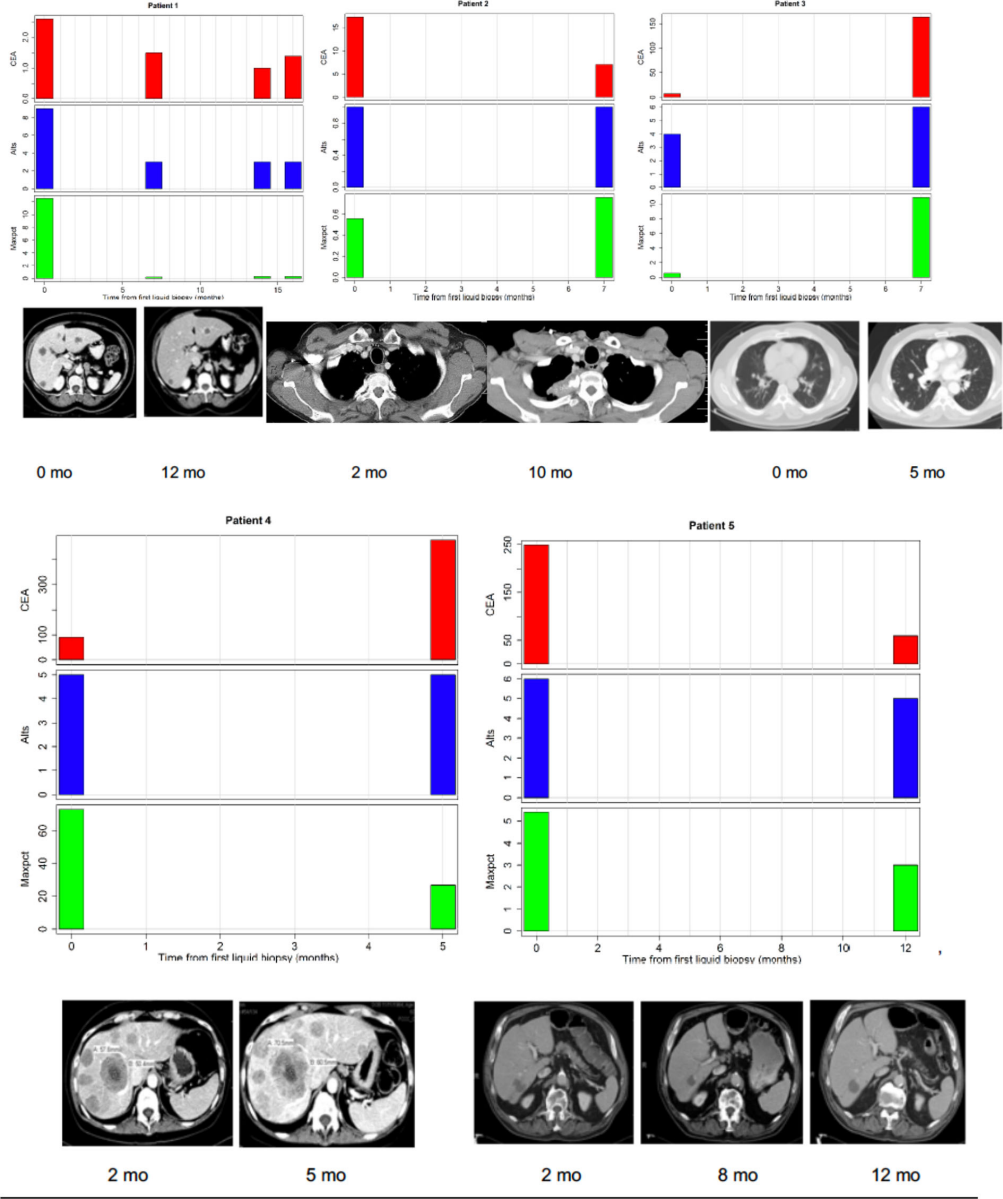
Correlations between tumor burden as assessed by radiographic imaging (CT scans) or CEA (tumor marker) and liquid biopsy mutation parameters (alts or number of alterations/number of mutated genes and maxpct or maximal allele frequency of mutated allele). (Top Left) Maxpct, CEA, and alts follow a downward trend as disease on CT scan improves. Center: With growth of mediastinal mass on CT, note rise in maxpct, CEA, and alts. (Top Right) As lung disease worsens on CT, maxpct, CEA, and alts increase. (Bottom Left) Despite increasing tumor on CT scan and rising CEA, maxpct did not rise. Liquid biopsy did contain APC and TP53 mutations, indicating presence of ctDNA. (Bottom Right) Liver metastases decreased between 1/2017 and 7/2017 and then increased in 11/2017. Allele freq. low (24%) probably due to low disease burden on CT.

